# The Actin Cytoskeleton at the Immunological Synapse of Dendritic Cells

**DOI:** 10.3389/fcell.2021.679500

**Published:** 2021-08-02

**Authors:** José Luis Rodríguez-Fernández, Olga Criado-García

**Affiliations:** Department of Cellular and Molecular Biology, Centro de Investigaciones Biológicas Margarita Salas, Consejo Superior de Investigaciones Científicas, Madrid, Spain

**Keywords:** dendritic cell, immunological synapse, actin, T cell activation, cytoskeleton

## Abstract

Dendritic cells (DCs) are considered the most potent antigen-presenting cells. DCs control the activation of T cells (TCs) in the lymph nodes. This process involves forming a specialized superstructure at the DC-TC contact zone called the immunological synapse (IS). For the sake of clarity, we call IS(DC) and IS(TC) the DC and TC sides of the IS, respectively. The IS(DC) and IS(TC) seem to organize as multicentric signaling hubs consisting of surface proteins, including adhesion and costimulatory molecules, associated with cytoplasmic components, which comprise cytoskeletal proteins and signaling molecules. Most of the studies on the IS have focused on the IS(TC), and the information on the IS(DC) is still sparse. However, the data available suggest that both IS sides are involved in the control of TC activation. The IS(DC) may govern activities of DCs that confer them the ability to activate the TCs. One key component of the IS(DC) is the actin cytoskeleton. Herein, we discuss experimental data that support the concept that actin polarized at the IS(DC) is essential to maintaining IS stability necessary to induce TC activation.

**Lord Carnarvon: “Can you see anything?”**

**Howard Carter: “Yes, wonderful things!”**

An exchange between wealthy patron Carnavon and archeologist Carter after the latter peered through a hole into Tutankhamun’s tomb.

## Introduction

Dendritic cells (DCs) are the most potent antigen-presenting cells (APCs; Banchereau and Steinman, 1998). There are two main groups of DCs: conventional and plasmacytoid (Banchereau and Steinman, 1998; Merad et al., 2013). DCs are found in tissues in the immature differentiation stage. In the presence of pathogens, they undergo a process of differentiation called maturation, which involves multiple phenotypical changes, including the upregulation of major histocompatibility complex class I (MHC-I) and class II (MHC-II) and costimulatory molecules, like CD80 and CD86. Mature DCs migrate to the lymph nodes (LNs), where they present pathogen-derived peptides *via* MHC-I to CD8^+^ T cells (TCs) or *via* MHC-II to CD4^+^ TCs, resulting in the activation of these lymphocytes. Hereafter, unless otherwise indicated, when we use the word DCs, we refer to the conventional mature DCs. Several studies have shown that activation of naïve TCs in the LNs involves, first, brief serial DC-TC encounters, which are antigen independent, followed by prolonged and stable antigen-dependent contacts that last several hours (Delon et al., 1998; Iezzi et al., 1998; Stoll et al., 2002; Bajenoff et al., 2003; Bousso and Robey, 2003; Mempel et al., 2004; Miller et al., 2004; Shakhar et al., 2005; Celli et al., 2007). Finally, the TCs recover their motility and proliferate (Mempel et al., 2004; Celli et al., 2007; Scholer et al., 2008). The region of tight adhesion that connects DCs and TCs when they establish stable interactions is called the immunological synapse (IS). We call the DC and TC sides of the IS the IS(DC) and IS(TC), respectively. Most studies on the IS have centered on the IS(TC), and analyses of the IS(DC) are sparse (Riol-Blanco et al., 2009; Rodriguez-Fernandez et al.,2010a,b; Benvenuti, 2016; Verboogen et al., 2016; Gomez-Cabanas et al., 2019; Alcaraz-Serna et al., 2021). Herein, we analyze the role of the filamentous-actin (F-actin) cytoskeleton of the IS(DC) in TC activation.

## Organization of the Plasma Membrane Proteins Components of the IS(DC)

The first studies on the IS focused on the IS(TC) (Monks et al., 1998; Grakoui et al., 1999). In one of the experimental models used, the CD4^+^ TCs were plated on a glass-supported lipid bilayer that was converted into a surrogate APC by inserting the intercellular adhesion molecule 1 (ICAM-1), the ligand of the integrin lymphocyte function-associated antigen (LFA-1), and peptides bound to MHC (pMHC; Grakoui et al., 1999). In another model, the CD4^+^ TCs were allowed to form IS with B cells (BCs; Monks et al., 1998; Grakoui et al., 1999). Following the binding of the TCs either to the glass-supported lipid bilayer or to the BCs, the costimulatory molecule CD28 and the TC receptor (TCR) clustered together in a region called the central supramolecular activation cluster (cSMAC). Contiguous to this region are found LFA-1 molecules that form a ring called peripheral SMAC (pSMAC). Large negatively charged molecules like CD43 and CD45 organize in an outermost ring called distal SMAC (dSMAC; Monks et al., 1998; Grakoui et al., 1999; Freiberg et al., 2002). Interestingly, when the DCs form the IS with TCs (naïve or activated) at the IS(TC), instead of the monocentric organization described above, surface proteins form multiple protein clusters that include TCRs, adhesion proteins, and costimulatory molecules (Brossard et al., 2005; Rothoeft et al., 2006; Reichardt et al., 2007; Fisher et al., 2008; Tseng et al., 2008; Thauland and Parker, 2010). The ability of the DCs to promote multicentric IS(TC) could contribute to explain why they are such potent APCs. Numerous clusters of CD3 and costimulatory molecules multiply the signaling from these receptors, resulting in robust TC activation (Leithner et al., 2021). Supporting this concept, TCs plated on surrogate-patterned APCs that promote TCR or CD28 clusters show enhanced functionality (Mossman et al., 2005; Shen et al., 2008).

## Polarization of F-Actin at the IS(DC)

The following examples, in which fixed cells were stained with phalloidin, show that F-actin polarizes in the IS(DC) upon allogeneic or antigen-specific DC-TC formation. Allogenic conjugates include (i) bone marrow-derived DCs (BM-DCs) (BALB/c genetic background) and CD4^+^ TCs (C57BL/6 genetic background) (Al-Alwan et al., 2001b) and (ii) human monocyte-derived DCs and allogeneic lymphoblasts (Riol-Blanco et al., 2009). Antigen-specific conjugates include (i) OVA peptide-loaded BM-DC, from BALB/c or C57BL/6 mice, and DO11.10 or OTII CD4^+^ TCs, respectively (Al-Alwan et al., 2003; Eun et al., 2006; Riol-Blanco et al., 2009), and (ii) OVA peptide-loaded BM-DCs and OTI CD8^+^ TCs (Tanizaki et al., 2010). A drawback of these fluorescence microscopy analyses performed with fixed conjugates is that it is difficult to know for certain whether the phalloidin-stained F-actin belongs to the IS(TC) or the IS(DC). However, recently, the use of Lifeact, an amino acid fragment of the protein ABP140 that binds selectively to F-actin, has solved this problem (Riedl et al., 2008; Leithner et al., 2021). High-resolution confocal microscopy analysis of Lifeact-green fluorescent protein (Lifeact-GFP)-expressing DCs that form IS with OTII CD4^+^ TCs shows that F-actin displays at the IS(DC) a multifocal organization, with foci of different sizes separated by regions where actin is sparse (Leithner et al., 2021). Finally, fluorescence recovery after photobleaching (FRAP) experiments performed with mCherry-labeled actin-transfected BM-DCs that interact with OTII TCs showed a slower recovery at the IS(DC) compared with the cortex, suggesting a higher stability and specific molecular features of the F-actin network at the IS(DC) (Malinova et al., 2016).

## Surface Proteins That Induce Actin Accumulation in the IS(DC)

Engagement of MHC-II, MHC-I, or LFA-1 with specific antibodies bound to polystyrene beads induces F-actin accumulation only in DCs that bind to beads associated with anti-MHC-II antibodies (Al-Alwan et al., 2003). The lack of effect of MHC-I was unexpected because F-actin accumulates at the IS(DC) in DC-OTI CD8^+^ TC conjugates (Tanizaki et al., 2010). Moreover, engagement of MHC-I on the membrane of endothelial cells with antibodies induces activation of the F-actin regulator ras homolog family member A (RhoA) (Coupel et al., 2004; Lepin et al., 2004) and actin organization (Lepin et al., 2004; Jin et al., 2007; Ziegler et al.,2012a,b). Hence, other experimental strategies, including different anti-MHC-I antibodies, should be used before ruling out that MHC-I controls F-actin accumulation in DCs. An analysis of wild-type (WT)-BM-DCs or CD80/86 knock-out (KO)-BM-DCs interacting with DO11.10 CD4 + TCs suggests that CD80/CD86 induces actin polarization at the IS(DC) (Rothoeft et al., 2006). However, actin failed to accumulate at the IS(DC) upon engagement of CD86 on DCs with antibodies or when BM-DCs, expressing that the CD28 receptors CD80 and CD86 interact with human Jurkat cells expressing murine CD28 (Al-Alwan et al., 2003; Rothoeft et al., 2006). Therefore, stimulation of CD80 or CD86 is not sufficient to promote F-actin aggregation. Finally, the semaphorin receptor Plexin-A1, which is localized at the IS(DC), can also induce RhoA activation and F-actin polarization in this region (Eun et al., 2006).

## Role of F-Actin and Actin-Regulatory Proteins at the IS(DC) on TC Activation

**FIGURE 1:**
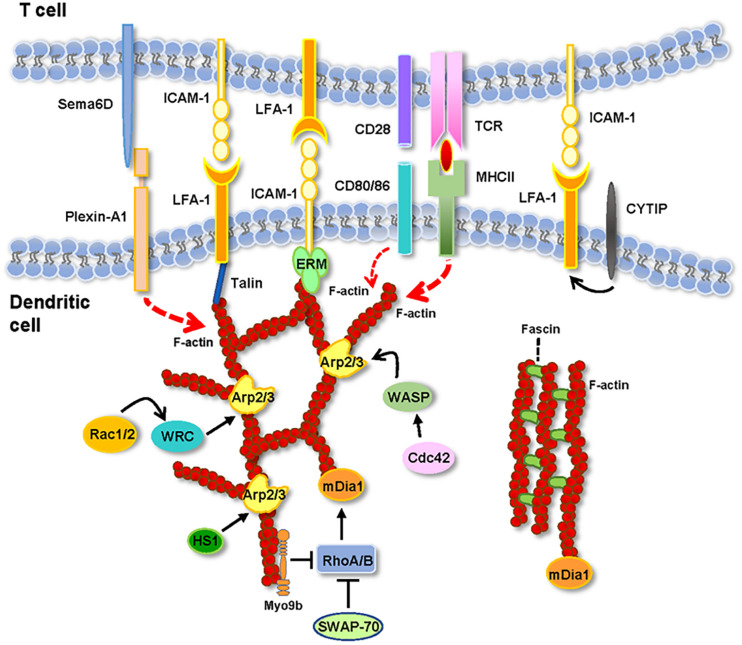
Actin and actin-regulatory proteins at the IS(DC). In the figure are depicted the molecules described in the text. See the text for details.

Below, we analyze reports that provide information on the role of DC’s F-actin and actin-regulatory proteins on TC activation (Figure 1 and Table 1). When analyzing these experimental data, it is important to take into consideration several points. First, the focus of most of the studies available on this issue was not the IS(DC). Second, the proteins analyzed can be expressed in the IS(DC) and elsewhere in DCs, like the DCs’ cortex (e.g., F-actin, WRC, WASP, and Myo9b), implying that these proteins may exert their regulatory effects inside and/or outside the IS(DC) (e.g., WASP, Rac1/2, and mDia also regulate migration). Third, actin-regulatory proteins can also govern actin-independent functions (e.g., HS1). Fourth, the experimental strategies employed to study the role of these molecules, namely, the use of pharmacological agents to inhibit F-actin or DCs deficient in actin-regulatory proteins, do not discriminate between the IS(DC) and other intracellular regions.

**TABLE 1:** Effects of the perturbation of F-actin and actin-regulatory molecules in the DCs. In the table are presented the effects observed following the interaction between TCs and either pharmacologically inhibited or KO-DCs, which are compared with the results obtained with TCs that interact with uninhibited or WT-DCs controls.

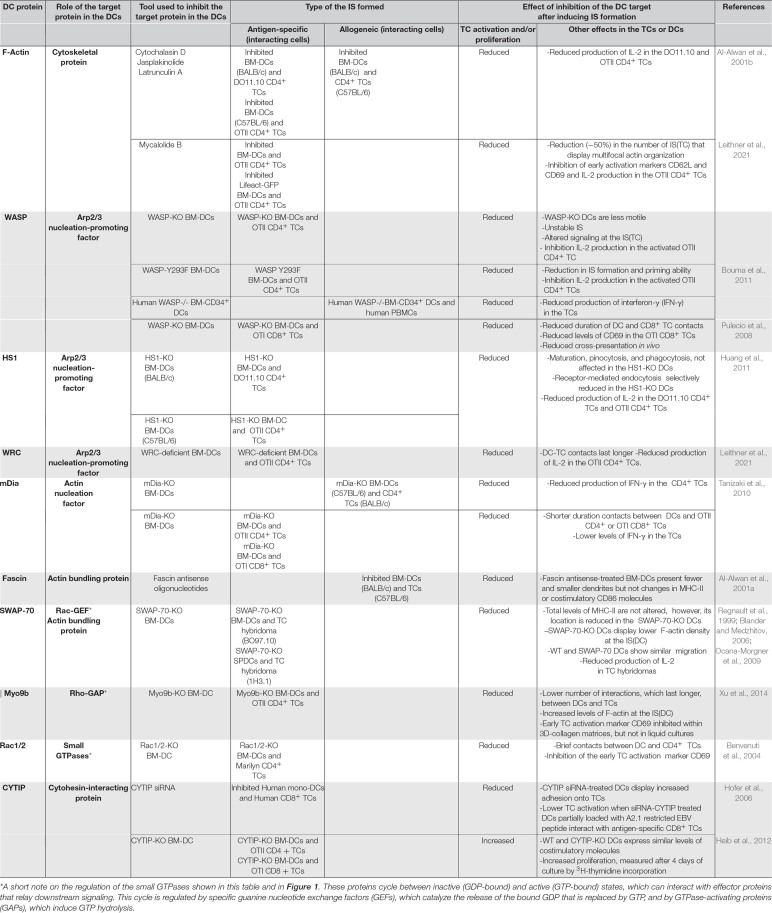

*A short note on the regulation of the small GTPases shown in this table and in Figure 1. These proteins cycle between inactive (GDP-bound) and active (GTP-bound) states, which can interact with effector proteins that relay downstream signaling. This cycle is regulated by specific guanine nucleotide exchange factors (GEFs), which catalyze the release of the bound GDP that is replaced by GTP, and by GTPase-activating proteins (GAPs), which induce GTP hydrolysis.

### Filamentous Actin

To analyze the role of F-actin at the IS(DC), pharmacological agents have been used that alter actin stability, including cytochalasin D, latrunculin A, and mycalolide B (MycB), which disrupt F-actin, and Jasplakinolide, which stabilizes it (Fenteany and Zhu, 2003). When DCs treated with any of these inhibitors interact with DO11.10 or OTII CD4^+^ TCs, the activation and proliferation of these lymphocytes is inhibited (Al-Alwan et al., 2001b; Leithner et al., 2021; Table 1). These results emphasize the importance of the integrity of the DCs’ actin cytoskeleton for TC activation (Al-Alwan et al., 2001b; Leithner et al., 2021). Confocal microscopic analyses of DCs that interact with Lifeact-GFP expressing OTII CD4^+^ TCs show that IS(TC) form multiple actin foci (Leithner et al., 2021). However, when DCs pretreated with MycB to disrupt F-actin were allowed to interact with the Lifeact-GFP OTII CD4^+^ TCs, ∼50% of the IS(TC) present a ring of F-actin surrounding an actin-free circle, instead of a multifocal actin organization (Leithner et al., 2021; Table 1). Hence, multifocal actin at the IS(DC) contributes partially to stabilizing multifocal actin at the IS(DC) and predictably also to the formation of the multicentric IS(TC) (Brossard et al., 2005; Rothoeft et al., 2006; Reichardt et al., 2007; Fisher et al., 2008; Tseng et al., 2008; Thauland and Parker, 2010), although this has to be confirmed in future studies because, in the experiments described, the authors did not stain the surface proteins, such as CD3, and other molecules, which organize in foci in the IS(TC) (Leithner et al., 2021). Finally, F-actin at the IS(DC) can also control DC-TC adhesion by selectively regulating the lateral mobility on the plasma membrane of ICAM-1 (Comrie et al., 2015). Immobilized ICAM-1 at the IS(DC) can promote LFA-1 activation on the IS(TC) and increase DC-TC adhesion (Feigelson et al., 2010).

### Wiskott–Aldrich Syndrome Protein

Wiskott–Aldrich syndrome protein (WASP) is a nucleation-promoting factor (NPF) that activates the actin-related protein 2/3 (Arp2/3) complex (Figure 1). Arp2/3 is an actin-nucleation factor (ANF) that assembles actin dimers or trimers that serve as nuclei that subsequently polymerize into Y-branched actin networks (Schonichen and Geyer, 2010). WASP organize in foci within the IS(DC) (Leithner et al., 2021). WASP-KO BM-DCs show reduced motility and lower F-actin levels (Bouma et al., 2011; Malinova et al., 2016). WASP-KO BM-DCs that interact with OTII CD4^+^ TCs *in vitro* present a high number of transient interactions and reduced DC-TC contact areas, suggesting that in the DCs, WASP may stabilize the interactions with the TCs (Bouma et al., 2011; Malinova et al., 2016; Table 1). Similar conclusions have been obtained in *in vitro* and *in vivo* studies that analyze the interactions between WASP-KO BM-DCs and OTI CD8^+^ TCs (Pulecio et al., 2008). At the IS(DC) formed by the WASP-KO BM-DCs, the levels of ICAM-1 and MHC-II are reduced (Malinova et al., 2016). The levels of TCR, LFA-1, F-actin, and talins are also reduced at the IS(TC). Moreover, TCR-dependent signaling was also altered, resulting in reduced IL-2 production, and TC proliferation (Bouma et al., 2011; Malinova et al., 2016). Further supporting a role for the WASP/Arp2/3 axis in IS formation, DCs that express Y293F-WASP (a mutation that impairs WASP’s ability to activate Arp2/3) display a low number of IS with TCs and reduced priming ability (Bouma et al., 2011). Finally, in FRAP experiments performed with Cherry-labeled actin-transfected WASP-KO, Y293F-WASP, and WT-BM-DCs that formed IS with OTII CD4^+^ TCs, actin recovery at the IS(DC) was slower in the WT DCs compared with the WASP-KO and Y293F-WASP DCs, suggesting that WASP-Arp2/3-mediated formation of branched actin stabilizes the actin network at the IS(DC) (Malinova et al., 2016).

### Hematopoietic Lineage Cell-Specific Protein 1

Hematopoietic lineage cell-specific protein 1 (HS1) is a NPF that induces Arp2/3-dependent branched actin networks, and, moreover, it can also bind and stabilize this network (Weaver et al., 2001; Uruno et al., 2003; Hao et al., 2005; Dehring et al., 2011; Figure 1). Since HS1 expression increases during DC maturation (Huang et al., 2011), it is interesting to study whether this molecule could regulate actin organization at the IS(DC) and DCs’ priming ability (Table 1). WT and HS1-KO BM-DCs bind and present OVA peptides equally as well with DO11.10 CD4^+^ TCs (Huang et al., 2011). However, HS1-KO BM-DCs loaded with intact OVA protein display a reduced ability to activate the CD4^+^ TCs (Huang et al., 2011). WT and HS1-KO BM-DCs present the MHC-I-restricted VSV8 peptide equally as well with the CD8^+^ TC hybridoma N15. However, when VSV8 was complexed with the protein GRP94, which also uses the MHC-I pathway of antigen presentation, the priming ability of the HS1-KO BM-DCs was impaired. It was observed that receptor-mediated endocytosis was selectively inhibited in the HS1-KO BM-DCs, preventing antigen uptake (Huang et al., 2011). It was also found that HS1 is required for antigen uptake because it participates, together with dynamin 2, in the scission of the endocytic vesicles (Huang et al., 2011). Hence, although HS1 is a NPF, it apparently regulates antigen presentation through the control of antigen endocytosis.

### WAVE Regulatory Complex

WASP-family verprolin homologous proteins (WAVE) regulatory complex (WRC) is an NPF that activates Arp2/3 and induces branched actin (Buracco et al., 2019; Figure 1). WRC is found in the IS(DC), but it also associates with F-actin at the DC cortex (Leithner et al., 2021). Upon interaction of WRC-deficient BM-DCs (Park et al., 2008) with OTII CD4^+^ TCs, F-actin displays a multifocal organization in the IS(TC), like the IS(TC) formed by the WT BM-DCs (Leithner et al., 2021; Table 1). However, F-actin levels at the IS(DC) are reduced, suggesting that WRC promotes actin accumulation in this region. WRC-deficient DC-TC contacts last longer and display larger areas of contact (Leithner et al., 2021). These prolonged interactions are associated with an increase in the levels of the phospho-ezrin-radixin-moesin (ERM), suggesting a higher anchoring of ICAM-1 to cortical F-actin, which may result in the immobilization of this ligand and increased LFA-1-mediated DC-TC adhesion (Comrie et al., 2015; Leithner et al., 2021). These abnormal long-lasting interactions between WRC-deficient DCs and TCs may explain the observed reduction in the activation of the TCs (Leithner et al., 2021).

### Mammalian Homolog of Diaphanous

Mammalian homolog of diaphanous (mDia1) is an ANF of the formin family that promotes F-actin elongation (Schonichen and Geyer, 2010; Figure 1). The mDia-KO-BM-DCs display reduced adhesion and impaired migration (Tanizaki et al., 2010). CD4^+^ TCs that establish alloreactive interactions with mDia-KO BM-DC also presented reduced proliferation and low interferon-γ (IFN-γ) production (Table 1). Two-photon microscopy analysis shows that mDia-KO BM-DCs that interact with OTII CD4^+^ TCs or with OTI CD8 + TCs establish brief contacts within the LNs, indicating that DCs’ mDia is important for keeping stable ISs (Tanizaki et al., 2010). Hence, correct TC activation requires of mDia1 expression in the DCs.

### Fascin

Fascin is an actin-bundling protein whose expression is increased during DC maturation (Mosialos et al., 1996; Al-Alwan et al., 2001a; Yamashiro, 2012). In mature DCs, fascin, which can localize to the IS(DC), controls dendrite formation (Al-Alwan et al.,2001a,b; Rothoeft et al., 2006; Figure 1). In antigen-specific models of IS formation, accumulation of fascin and F-actin correlates with more extended contacts between DCs and TCs, increased TC proliferation, and CD4^+^ Th1 TC-dependent responses (Rothoeft et al., 2006). Using an allogeneic model of IS formation, it is observed that the levels of fascin in DCs correlate with the ability of these cells to stimulate the TCs (Al-Alwan et al., 2001a; Table 1). Finally, in an alloreactive IS model, it was observed that a reduction of fascin levels in the BM-DCs with antisense oligonucleotides inhibits their ability to allostimulate the TCs (Al-Alwan et al., 2001a). Therefore, fascin-mediated bundling of F-actin in DCs contributes to TC priming.

### Switch-Associated Protein 70

Switch-associated protein 70 (SWAP-70) is a Rac GEF (see Table 1 and legend) that also controls F-actin bundling (Gomez-Cambronero, 2012; Chacon-Martinez et al., 2013; Figure 1). Although WT and SWAP-70-KO DCs express similar total MHC-II levels, SWAP-70-KO BM-DCs show a reduced expression of MHC-II on the plasma membrane (Ocana-Morgner et al., 2009). OVA peptide-loaded SWAP-70-KO DCs’ ability to prime TCs is impaired (Ocana-Morgner et al., 2009), as shown by their reduced ability to activate two different MHC-II-restricted TC hybridomas (Regnault et al., 1999; Blander and Medzhitov, 2006). In SWAP-70-KO DCs, the actin regulatory GTPases RhoA and RhoB are constitutively activated, resulting in an increase in the amount of F-actin in these cells (Ocana-Morgner et al., 2009; Sit and Manser, 2011). Inhibition of RhoA and RhoB in the SWAP-70-KO DCs with *Clostridium botulinum* increased MHC-II on their plasma membrane and helped recover their ability to activate the TCs. Hence, it is suggested that the high F-actin levels prevent the correct MHC-II localization on the plasma membrane (Bretou et al., 2016). Therefore, SWAP-70 may inhibit RhoA and RhoB activation, which prevents an abnormal increase in F-actin and allows MHC-II localization on the membrane of the DCs (Ocana-Morgner et al., 2009).

### Myosin IXb

Myosin IXb (Myo9b) is a cytoskeletal motor that displays Rho-GTPase-activating protein (GAP) activity (see Table 1 and legend). Myo9b colocalizes with F-actin in DCs (Hanley et al., 2010; Xu et al., 2014; Figure 1). Compared to WT-BM-DCs, KO-Myo9B BM-DCs present a low number interaction with OTII CD4^+^ TCs, although these interactions last longer (Xu et al., 2014). F-Actin is highly increased in the IS(DC) of the KO-Myo9B BM-DCs that form IS with OTII CD4^+^ TCs. However, KO-Myo9B BM-DC-OTII CD4^+^ TCs’ interactions resulted in reduced proliferation within 3D-collagen matrices but not in liquid co-cultures (Xu et al., 2014). These results could be due to the different spatiotemporal organization of F-actin in the IS(DC) of the KO-Myo9B DCs under both conditions (Xu et al., 2014).

### RhoA, Rac1 and Rac 2

RhoA, Rac1, and Rac 2 belong to the Rho GTPase subfamily, which are critical regulators of the actin cytoskeleton (see Figure 1, and Table 1 and legend) (Sit and Manser, 2011). Treatment of the DCs with epidermal cell differentiation inhibitor (EDIN) toxin, which inactivates RhoA, or inhibition of its downstream target, the Rho-associated protein kinase (ROCK), with Y27632, failed to affect IS formation between BM-DCs and OTII CD4^+^ TCs (Benvenuti et al., 2004). These results suggest that the effects of knocking down SWAP-70 on F-actin discussed above could be mediated by RhoB, instead of RhoA. DCs deficient in Rac1 and Rac2 show alterations in the F-actin organization, resulting in the absence of dendrites and reduced motility. *In vitro* analyses show that Rac1/2-KO BM-DCs do not form stable contacts with CD4^+^ TCs. Consistent with these results, the Rac1/2-KO BM-DCs show a reduced ability to activate OTII CD4^+^ TCs. It was suggested that this inhibition was due to deficient actin dynamics in the Rac1/2-KO DCs that prevent these cells from engulfing and establishing full contacts with TCs (Benvenuti et al., 2004).

### Cytohesin-Interacting Protein (CYTIP)

Although CYTIP is not an actin-regulatory protein, we have included it in this review because it regulates LFA-1, which is an important molecule at the IS(DC) (Figure 1). In resting DCs, LFA-1 remains on the plasma membrane in an inactive state; that is, it cannot bind to its ligand ICAM-1. DCs express cytohesin-1, which interacts with the cytoplasmic β-subunit of LFA-1, resulting in its activation and binding to ICAM-1. CYTIP binds to cytohesin-1, which translocates from the membrane to the cytoplasm, leaving LFA-1 inactivated. During DC maturation, CYTIP levels increase and localize to the IS(DC) (Hofer et al., 2006). Studies on CYTIP in DCs are controversial (Table 1). In experiments in which CYTIP was reduced with siRNA in human DCs (Hofer et al., 2006), these cells showed a diminished ability to induce proliferation of autologous antigen-specific CD8^+^ TCs (Hofer et al., 2006). Other studies show that knocking down CYTIP with siRNA in BM-DCs extends antigen-specific contacts with OTII CD4^+^ TCs or OTI CD8^+^ TCs and reduces the activation and proliferation of these cells (Balkow et al., 2010). In contrast, in experiments performed with DCs obtained from CYTIP KO mice (Coppola et al., 2006), CYTIP KO BM-DCs enhanced antigen-specific activation OTI and OTII TCs (Heib et al., 2012).

## Concluding Remarks

The study of the role of F-actin at the IS(DCs) is at its inception. The data discussed above suggest that F-actin polarization at the IS(DC) maintain IS stability necessary to induce TC activation. A network of actin-regulatory proteins controls F-actin organization at the IS(DC) (Figure 1). The pharmacological disruption of F-actin, the knockdown of Rac1/2, or the increase of F-actin levels after knocking down Myo9b reduce the ability of the DCs to activate the TCs. Knockdown of WASP or WRC, which promotes branched actin through Arp2/3, or mDia, which regulates linear F-actin, or fascin, a bundling protein, results in inhibition of TC activation. Deletion of WASP, mDia, fascin, and Rac1/2 reduces the number and/or the duration of DC-TC contacts. In contrast, knockdown of WRC or Myo9b results in extended DC-TC contacts and inhibition of TC activation. In summary, perturbation of F-actin dynamics at the IS(DC) leads to the inhibition of TC activation. Multiple aspects of the F-actin regulation and functions at the IS(DC) need to be addressed in the future. For this purpose, it is very important to develop experimental strategies that selectively target F-actin and its regulatory proteins at the IS(DC). Many wonderful things remain to be discovered at the IS(DC).

## Author Contributions

JR-F designed the work and wrote the manuscript with the help of OC-G. OC-G prepared the table and the figure. Both authors contributed to manuscript revision, and read and approved the submitted version.

## Conflict of Interest

The authors declare that the research was conducted in the absence of any commercial or financial relationships that could be construed as a potential conflict of interest.

## Publisher’s Note

All claims expressed in this article are solely those of the authors and do not necessarily represent those of their affiliated organizations, or those of the publisher, the editors and the reviewers. Any product that may be evaluated in this article, or claim that may be made by its manufacturer, is not guaranteed or endorsed by the publisher.
